# Design and optimization of silymarin loaded in lyophilized fast melt tablets to attenuate lung toxicity induced *via* HgCl_2_ in rats

**DOI:** 10.1080/10717544.2022.2068696

**Published:** 2022-04-26

**Authors:** Nermin M. Sheta, Sylvia A. Boshra, Mohamed A. Mamdouh, Khaled M. Abdel-Haleem

**Affiliations:** a Pharmaceutics Department, Faculty of Pharmacy, October 6 University, Giza, Egypt; b Biochemistry Department, Faculty of Pharmacy, October 6 University, Giza, Egypt

**Keywords:** Silymarin, fast melt tablets, lyophilization, lung toxicity, miRNA 133a, miRNA155, COX-2

## Abstract

The present study aimed to develop fast melting tablets (FMTs) using silymarin (SM) owing to FMTs rapid disintegration and dissolution. FMTs represent a pathway to help patients to increase their compliance level of treatment *via* facile administration without water or chewing beside reduction cost. One of the methods for FMTs formulation is lyophilization. Optimization of SM-FMTs was developed via a 3^2^ factorial design. All prepared SM-FMTs were evaluated for weight variation, thickness, breaking force, friability, content uniformity, disintegration time (DT), and % SM released. The optimized FMT formula was selected based on the criteria of scoring the fastest DT and highest % SM released after 10 min (Q_10_). Optimized FMT was subjected to Fourier transform infrared spectroscopy (FT-IR), X-ray powder diffraction (XRD), and scanning electron microscopy (SEM) besides investigating its lung-protective efficacy. All SM-FMT tablets showed acceptable properties within the pharmacopeial standards. Optimized FMT (F7) scored a DT of 12.5 ± 0.64 Sec and % SM released at Q_10_ of 82.69 ± 2.88%. No incompatibilities were found between SM and excipients, it showed a porous structure under SEM. The optimized formula decreased cytokines, up-regulated miRNA133a, and down-regulated miRNA-155 and COX-2 involved in the protection against lung toxicity prompted by HgCl_2_ in a manner comparable to free SM at the same dosage.

## 1. Introduction

Silymarin (SM) is a yellowish powder extracted from the seeds of silybum marianum which contain several flavonoids namely, silybinin (highly active component), isosilybinin, silydianin, and sillychristin (Parveen et al., 2015; Surai, 2015; Gillessen & Schmidt, 2020; Song et al., 2021). SM possesses various pharmacological impacts, e.g., hepatoprotective, antihyperlipidemic, antitumor, antioxidant, anti-diabetic, cardio-protective, and anti-platelet activities (Rahimi et al., 2018; Taleb et al., 2018). Nevertheless, the pharmacological efficacy of SM is substantially low owing to its low water solubility (0.04 mg/mL), hence its dissolution turns out to be an absorption rate-limiting step (Sheta et al., 2020).

New SM dosage forms and preparation techniques concentrate on enhancing its efficacy via solving two drawbacks of SM namely, first-pass metabolism and low water solubility that may hinder SM biological efficacy. This can be achieved *via* developing an oral delivery system that allows pre-gastric absorption in addition to applying solubility improvement, hence enhancing the oral bioavailability of SM (Wen et al., 2008; Zhu et al., 2013; Tawfeek et al., 2018). FMTs have been developed as an appealing alternative to overcome the drawbacks of conventional SM oral tablets, such as the difficulty of swallowing in geriatrics by rapid disintegration in the mouth (30 Sec) and the faster onset of action, which leads to improved patient compliance, as well as bypassing first-pass metabolism (ElMeshad et al., 2020; Fouad et al., 2020). Several techniques are currently used to formulate FMT, including lyophilization, solid dispersion, direct compression, and molding (Bhowmik et al., 2009).

Several solubilization techniques have been proposed to enhance the solubility of SM, for instance, the development of liquisolid system and nanomicelles for improving oral absorption (Piazzini et al., 2019; Sheta et al., 2020) and the encapsulation of SM in bilosomes for improving its hepatoprotective activity (Mohsen et al., 2017). Based on the sales value and volume, the lyophilization technique has been the most successful for the formulation of FMTs (Badgujar & Mundada, 2011). The formulation of lyophilized FMT depends on the creation of a porous matrix made by subliming the water from the pre-frozen aqueous formulation of the drug matrix forming agents (water-soluble polymers) and other excipients, such as fillers and lyoprotectants (a substance added to prevent damage during lyophilization process) (ElMeshad et al., 2020). The water-soluble polymers used in this study were pullulan, gellan gum, and alginates besides matrix-supporting/disintegration-enhancing agents such as Pearlitol® SD and glycine as lyoprotectant (ElMeshad et al., 2020).

Pulmonary fibrosis is a condition marked by complex inflammatory processes that lead to excessive fibroblast proliferation and progressive connective tissue deposition in the pulmonary parenchyma (Crestani et al., 2007). The disease induces a significant decline in lung function, as well as restricting symptoms and causes a poor quality of life. Unfortunately, considering the seriousness of the condition, existing pulmonary fibrosis therapies are of limited efficacy and have severe side effects. Mercury is the third most harmful heavy metal, posing significant health hazards due to its toxicity (Othman et al., 2014). It is widely distributed in certain occupational settings and causes major damage to different organs due to unintentional and/or occupational exposures (Durak et al., 2010).

Antifibrotic effects of Silibinin have been shown in animal and in vitro models (Kim et al., 2012). There is strong preclinical evidence through *in vitro* and *in vivo* models that SM (particularly, the silibinin flavonoid components) inhibit cancer promotion and progression. In preclinical studies, SM proved to have direct anticancer activity against prostate, breast, and ectocervical carcinoma cells (Pradhan and Girish, 2006). Preclinical studies by (Kren and Walterova, 2005) found that SM decreased myeloperoxidase (MPO) activity, tumor necrosis factor-alpha (TNF-α), and interleukin 6 (IL-6) levels in colon tissues, thereby improving the histopathological characteristics. The present study aimed at the formulation and optimization of SM-loaded FMT to avoid the first-pass effect and improve drug solubility then evaluate its therapeutic efficacy through the investigation of its lung-protective efficacy after mercuric chloride-induced lung toxicity in rats.

## 2. Materials and methods

### 2.1. Materials

Silymarin was a free gift sample from CID company (Egypt); Pearlitol^®^ SD was obtained from Roquette Frères Ltd. (Beinheim, France); glycine, potassium dihydrogen phosphate, disodium hydrogen phosphate, and mercuric chloride (Sigma-Aldrich, USA); Pullulan was a kind gift from Hayashibara Co., Ltd. (Okayama, Japan); protacid F120NM alginic acid and gellan gum (Kelcogel^®^) was purchased from MP Biomedical (France).

### 2.2. Methods

#### 2.2.1. Construction of factorial design

FMTs containing SM were formulated and optimized via outcomes adopted from 3^2^ full factorial designs using Design Expert^®^ software version 11 (Stat-Ease, Inc., Minneapolis, MN, USA). This design involved studying the effect of two independent variables, namely, X_1_: binder type and X_2_: binder concentration on disintegration time (DT) in Sec (Y_1_), and % drug released after 10 min (Q_10_) (Y_2_) which were selected as the dependent variables. Nine possible runs were prepared according to the pre-mentioned design, where the variables and levels are shown in Table 1.

**Table 1.** Full factorial design (3^2^) for preparation of SM FMT.

Independent variables	Levels		
X_1_: binder type	Gellan gum	Protacid	Pullulan
X_2_: binder concentration	15%	30%	45%
Dependent variables	Desirability constraints		
*Y*_1_: DT (Sec)	Minimize		
*Y*_2_: % SM release (*Q*_10_)*	Maximize		

*Q_10_ is the comparison of % SM released at 10 minutes.

#### 2.2.2. Preparation of FMT by lyophilization

The specified amount of binder, according to the composition shown in (Table 2) was dispersed in 2 mL of distilled water (w/w) with the temperature maintained at 40 °C using a hot plate with a magnetic stirrer (MS-300HS, Korea) until a clear solution was attained. Calculated amounts of perlitol and glycine were then added to the previous mixture and stirred until completely dissolved. An accurately weighed amount of SM was dispersed in the above mixture to obtain a dose of 70 mg. The resulted solution was poured into the pocket of the tablet blister (2 cm diameter), which was then kept at −20 °C for 24 h (Moutasim et al., 2017). The frozen tablet was then placed in lyophilizer (Christ Alpha 1-2LD plus, Munich Germany) with condenser temperature at −55 °C and vacuum at 7.6 Pa for 24 h for complete dryness. The regime for lyophilization cycle involved primary drying at −30 °C and 0.37 mbar for 12 h followed by 12 h of secondary drying at 20 °C and 0.01 mbar (ElMeshad et al., 2020).

**Table 2.** The composition of FMTs containing SM.

Formula code	Lyophilized FMTs ingredients (mg)					
	SM	Perlitol	Gellan gum	Protacid	Pullulan	Glycine
F1	70	80	30	–	–	20
F2	70	50	60	–	–	20
F3	70	20	90	–	–	20
F4	70	80	–	30	–	20
F5	70	50	–	60	–	20
F6	70	20	–	90	–	20
F7	70	80	–	–	30	20
F8	70	50	–	–	60	20
F9	70	20	–	–	90	20

Total weight of one FMT = 200 mg.

#### 2.2.3. Characterization of the prepared FMTs

##### 2.2.3.1. Weight variation

This test was performed on twenty tablets according to the US Pharmacopeia to ensure the uniformity of weight (United States Pharmacopeia 24/NF19, 2000; ElMeshad et al., 2020).

##### 2.2.3.2. Thickness

Tablet thickness was obtained *via* measuring the thickness at the center of the formulated tablet via a calibrated dial caliper. Ten tablets of each formula were selected randomly, and their thicknesses were measured (Teaima et al., 2021).

##### 2.2.3.3. Breaking force

Breaking force test was carried out on three tablets of each formula by using a tablet hardness tester Monsanto Type (Model HT-50P, Thermonik, Campbell Electronics, Veer Savarkar Marg, Mumbai). The tablets were placed in the space provided on the device with a horizontal position. The number shown on the digital screen when the tablet broke was recorded as the breaking force of the tablet where the mean was calculated from three readings for each FMT (ElMeshad et al., 2020; Teaima et al., 2021).

##### 2.2.3.4. Friability test

An amount of 6.5 g of total tablet weight of each formula (W1) (initial weight) (Goel et al., 2009; ElMeshad et al., 2020) placed into the friability tester which was rotated for 4 min at a speed of 25 rpm. Once the cycle was accomplished, the tablets were removed, cleaned from dust, and weighed again (W2) (final weight). Friability % should be not more than 1% (ElMeshad et al., 2020) and can be calculated from the formula below:

$$\text{Friability\% }=\;[\frac{W1-W2\;(\text{Loss of weight})}{W1\;(\text{Initial weight})}]\times \;100$$

##### 2.2.3.5. Content uniformity

FMTs containing SM were dissolved in 500 mL of simulated saliva fluid (SSF) and stirred until completely dissolved (Tafere et al., 2021). Drug content was estimated spectrophotometrically at the predetermined maximum wavelength (*λ*_max_ = 285.8 nm) (Sonali et al., 2010). The experiment was done in triplicates for each formula, and the mean value was determined.

##### 2.2.3.6. In-vitro disintegration time

FMTs disintegration time was determined by introducing each tablet formula into 900 mL SSF at 37 ± 0.5 °C *via* disintegration tester apparatus (Pharma test, Hainburg, Germany). The DT was defined as the time required for complete disintegration of the tablet with no remaining solid residue (Moutasim et al., 2017).

##### 2.2.3.7. In-vitro SM release from FMTs

SM FMTs dissolution was proceeded via the USP dissolution system, Distek (Model 2500i Type II, TCS-0500 Scheduler, New Jersey, USA) at 37 ± 0.5oC and at 50 rpm using 900 mL of SSF as the dissolution medium (Moqbel et al., 2016; Shohin et al., 2016; Fouad et al., 2020; AlAli et al., 2021; Ali et al., 2021. Samples were taken at (2, 4, 6, 8, 10, 20 and 30 min), 5 mL aliquots samples were withdrawn and instantaneously replaced with 5 mL SSF kept at the same temperature (Cirri et al., 2005; Sheta & Boshra, 2021). The samples were analyzed spectrophotometrically at the predetermined λmax = 285.8 nm. The experiment was repeated three times, and the mean value in each FMT formula was determined.

#### 2.2.4. Selection of the optimized formula

F7 was chosen as the optimal formula based on the factorial design results since it had the lowest DT and the most prominent drug release at Q_10_ with a desirability value of = 0.987, and hence it was further subjected to the following studies:

##### 2.2.4.1. Fourier transform infrared spectroscopy (FT-IR)

The spectra for pure SM, plain F7 and F7 were done separately by blending each sample with KBr at a ratio of 2:200, and the samples were directly loaded into FT-IR (IRAffinity-1, Shimadzu, Japan) (ElMeshad et al., 2020; Naveen et al., 2020; Sheta et al., 2020; Abdelmonem et al., 2021; Song et al., 2021) in the frequency range 4500-500 cm^−1^.

##### 2.2.4.2. X-ray powder diffraction (XRD)

The X-ray was obtained using an Advanced Diffraction system (Scintag Inc., USA) with a copper target at a voltage of 40 kV and intensity of 30 mA at a scanning speed of 1° C/min. The angle of diffraction gives an indication of the crystalline or amorphous nature of the main active constituent. XRD patterns were determined for pure SM, plain F7, and F7 (Naveen et al., 2020; Sheta et al., 2020).

##### 2.2.4.3. Scanning electron microscopy (SEM) visualization

Topographic visualization via SEM was performed for the pure SM and the optimum FMT F7 to determine the porous structure of the tablet and examine the morphology of sectioned surfaces. FMT F7 was cut transversely, and the internal matrix texture was examined at a voltage of 20 kv. Cross-section samples were prepared by cutting a thin slice of the FMT using a scalpel (Alejandro et al., 2020; ElMeshad et al., 2020; Naveen et al., 2020; Sheta et al., 2020).

#### 2.2.5. Investigation of lung-protective efficacy of SM and F7 in mercuric chloride induced lung toxicity in rats

##### 2.2.5.1. Experimental design and animals

Male albino rats (195 ± 10 g) were kept at a temperature of 22 ± 1 °C and a humidity of 55–60% in a light-controlled environment for one week to acclimatize and were given a standard diet and free access to water, and then they were distributed into 4 groups, each of 10 rats.

Group I: rats received 5 mL distilled water for 15 days.

Group II: rats received mercuric chloride (1 mg/kg) orally by gavage administration (Sozme et al., 2014) for 15 days.

Group III: rats received mercuric chloride (1 mg/kg) + F7 (100 mg SM/kg) for 15 days; F7 tablets were cut into pieces and allocated in the oral cavity of the rats by using forceps followed by administration of 20-50 microliters of water to enhance disintegration (Abdelmonem et al., 2021).

Group IV: rats received mercuric chloride (1 mg/kg) + free SM (100 mg/kg) orally after dissolution by using gavage administration for 15 days (Zhao et al., 2013).

Free SM dispersed in water was administered orally *via* a gavage tube which was inserted into the mouth then the tube was gently advanced along the upper palate until it reaches the esophagus, the material was administered using a syringe attached to the end of the tube. After dosing, the tube was removed gently at the same angle as insertion and the animal was returned to its cage. On day 16, the Blood of rats was collected using a 23-gauge needle from the lateral saphenous vein. Blood flow is stopped by applying pressure with sterile gauze to achieve hemostasis then the plasma was separated.

##### 2.2.5.2. Biochemical assays

To measure plasma TNF-α, TGF-β and IL-6 in addition to lung caspase 3 and monocyte chemoattractant protein-1 (MCP-1) levels, ELISA kits (Hengyuan Bio-technology Development Co., Ltd. Shanghai, China) and (Biolegend Systems, San Diego, CA, USA) were used. The animals were sedated with carbon dioxide prior to cervical dislocation; the procedure was as follows: the rat was placed in a normal standing position on a firm, flat surface and firmly grasped the base of the tail with one hand then the back of the neck at the base of the skull was pressed with the thumb and first finger of the other hand. The effectiveness of dislocation was confirmed by feeling for cervical tissue separation. This procedure followed the guidelines for the use of cervical dislocation for rodent euthanasia of the University of Texas at Austin institutional animal care and use committee.

##### 2.2.5.3. Determination of COX-2 using western blot

Lung samples of the rats from each group were obtained as follows: the lung tissues were cut into pieces, then stainless steel beads were added, followed by buffer (2 volumes of buffer for every mass of tissue) into a microcentrifuge, the tubes were placed into the bullet blender, after the run, the tubes were removed from the instrument and samples were inspected. This procedure followed the protocol for lung/tracheal tissue homogenization in the bullet blender by Scientific Instrument Services Inc.^TM^.

Sodium dodecyl sulfate – polyacrylamide gel electrophoresis (SDS-PAGE) was used to isolate the proteins and then transferred to polyvinylidene difluoride (PVDF) membranes. After being blocked in 5% milk- tris buffered saline (TBS) solution, membranes were incubated with the following primary antibodies: anti-COX-2 (Cayman Chemical, cat #160126) and anti-b-actin (Sigma Aldrich, cat # A5441). Incubation with sufficient HRP-conjugated secondary antibodies and chemiluminescent reagent (GE-Amersham) culminated in the visualization of protein bands.

##### 2.2.5.4. Determination of miRNA133a and miRNA155 using qRT-PCR

Total RNA from 200 µg tissue was isolated using mirVana PARIS Kit (Ambion, Austin, TX, USA) according to the manufacturer’s protocol. In order to normalize sample-to-sample variation, each sample was combined with denaturing solution (Ambion, Austin, TX, USA) before being spiked with synthetic C. elegans miRNAs (133a and 155) (5 nM/L, RiboBio, Guangzhou, China). To isolate complete RNA from tissue samples, the manufacturer's instructions were followed, and TRIzol (Invitrogen, Carlsbad, CA, USA) was used. Total RNA was eluted with 100 μl RNase-free water and kept at −80 °C until further analysis. The Nanodrop 2000 spectrophotometer (NanoDrop Technologies, Wilmington, DE, USA) was used to determine RNA concentration and purity.

##### 2.2.5.5. qRT-PCR of miRNAs 133a and 155

The expression levels of miRNAs 133a and 155 were evaluated using SYBR Green (SYBR® Premix Ex Taq™ II, TaKaRa, Dalian, China). The Bulge-LoopTM miRNA qRT-PCR Primer Package (RiboBio, Guangzhou, China), which contains basic reverse transcription (RT) and PCR primers, was used to amplify an individual miRNA (Zhu et al., 2017). RT reactions were carried out at 42 °C for 60 min, followed by 70 °C for 10 min, and qRT-PCR was carried out at 95 °C for 20 Sec, after 40 cycles, of 95 °C for 10 Sec, 60 °C for 20 Sec, and 70 °C for 10 Sec on a Light Cycler^®^ 480 Real-Time PCR System (Roche Diagnostics, Mannheim, Germany) in 384-well plates (Zhu et al., 2017).

All samples were tested three times. The specificity of PCR products was determined using melting curve analysis. The expression levels of both miR-133a and miR-155 were determined using a reverse transcription kit (Takara Bio USA, Inc.) as directed by the manufacturer's instructions by quantitative PCR (miRNA and mRNA) with TB green staining. The relative expression was calculated (Livak and Schmittgen, 2001), and the expression of GAPDH served as the internal control. The primers for miR-133a and miR-155 detection were created by Shanghai Sangon Biotech. The following primer sequences were used for PCR:

- miRNA-133a, forward 5′-GCACTGATGTGAGCTGCAAG-3′,
- reverse 5′-TTCATGAAGCTTTTAAGAAACATCTT-3′;
- miRNA-155, forward 5′-CGGCGGTTAATGCTAATTGTGAT-3′,
- reverse 5′-GTGCAGGGTCCGAGGT-3′;
- GAPDH forward, 5′-CATGAGAAGTATGACAAC​AGC​CT-3',
- reverse, 5'-AGT​CCTTCC​ACGATA​CCA AAGT-3'.

##### 2.2.5.6. Histological assessment

The lung of each rat was sliced, and sections were preserved in a 10% buffered formaldehyde solution for histological investigation. Sections of 5 µm in thickness were prepared and then stained with hematoxylin and eosin for light microscopy analysis (Bancroft and Steven, 1983).

## 3. Results and discussion

### 3.1. Characterization of the prepared FMTs

#### 3.1.1. Weight variation, thickness, breaking force, friability test and content uniformity

Table 3, reveals the results of characterization tests of SM FMTs. The mean weight and thickness of tablets ranged from 193 ± 0.006 to 201 ± 0.001 mg and 6.90 ± 0.12 to 7.30 ± 0.00 mm, respectively, indicating uniformity within the respective group of FMTs formulae. Regarding friability studies, all the prepared FMTs showed acceptable percentage weight loss ranging from 0.26 ± 0.09 to 0.62 ± 0.08%, which complies with the pharmacopeial standards indicating good mechanical resistance and durability of the tablets (ElMeshad et al., 2020). The breaking force of SM FMTs ranged from 1.26 ± 0.16 to 1.95 ± 0.13 kg/cm^2^, which falls within the pharmacopeial limit (United States Pharmacopeia 24/NF19, 2000; ElMeshad et al., 2020). The breaking force of FMTs was obviously influenced by the binder type (*p* < .0001) and binder concentration (*p* < .001) with a dominant effect of binder concentration as evident by its high sum of squares (0.7377 for binder concentration and 0.09164 for binder type).

**Table 3.** Characterization test outcomes for FMTs.

Formula	Mean weight (mg)	Thickness (mm)	Breaking force (kg/cm2)	Friability (%)	Drug content (%)	DT (Sec.)
F1	199.00 ± 0.002	7.10 ± 0.01	1.45 ± 0.15	0.53 ± 0.11	98.51 ± 0.68	19.00 ± 0.25
F2	201.00 ± 0.001	7.30 ± 0.00	1.54 ± 0.19	0.40 ± 0.13	99.35 ± 0.74	23.00 ± 0.19
F3	199.00 ± 0.005	7.00 ± 0.01	1.95 ± 0.13	0.26 ± 0.09	100.21 ± 0.48	28.00 ± 0.33
F4	193.00 ± 0.006	7.25 ± 0.02	1.35 ± 0.11	0.59 ± 0.07	99.47 ± 1.32	17.00 ± 0.78
F5	195.00 ± 0.001	7.00 ± 0.01	1.59 ± 0.27	0.37 ± 0.06	98.63 ± 0.41	22.00 ± 1.22
F6	197.00 ± 0.009	6.90 ± 0.12	1.74 ± 0.21	0.33 ± 0.09	99.18 ± 0.54	25.00 ± 0.85
F7	200.00 ± 0.002	6.95 ± 0.05	1.26 ± 0.16	0.62 ± 0.08	96.27 ± 1.98	12.50 ± 0.64
F8	196.00 ± 0.002	7.00 ± 0.02	1.33 ± 0.21	0.43 ± 0.05	99.34 ± 0.93	16.00 ± 1.05
F9	197.00 ± 0.005	7.20 ± 0.11	1.41 ± 0.08	0.39 ± 0.07	100.12 ± 0.84	21.00 ± 0.88

Data expressed as a mean value ± S.D.

#### 3.1.2. *In-vitro* disintegration time

**Figure 1.**
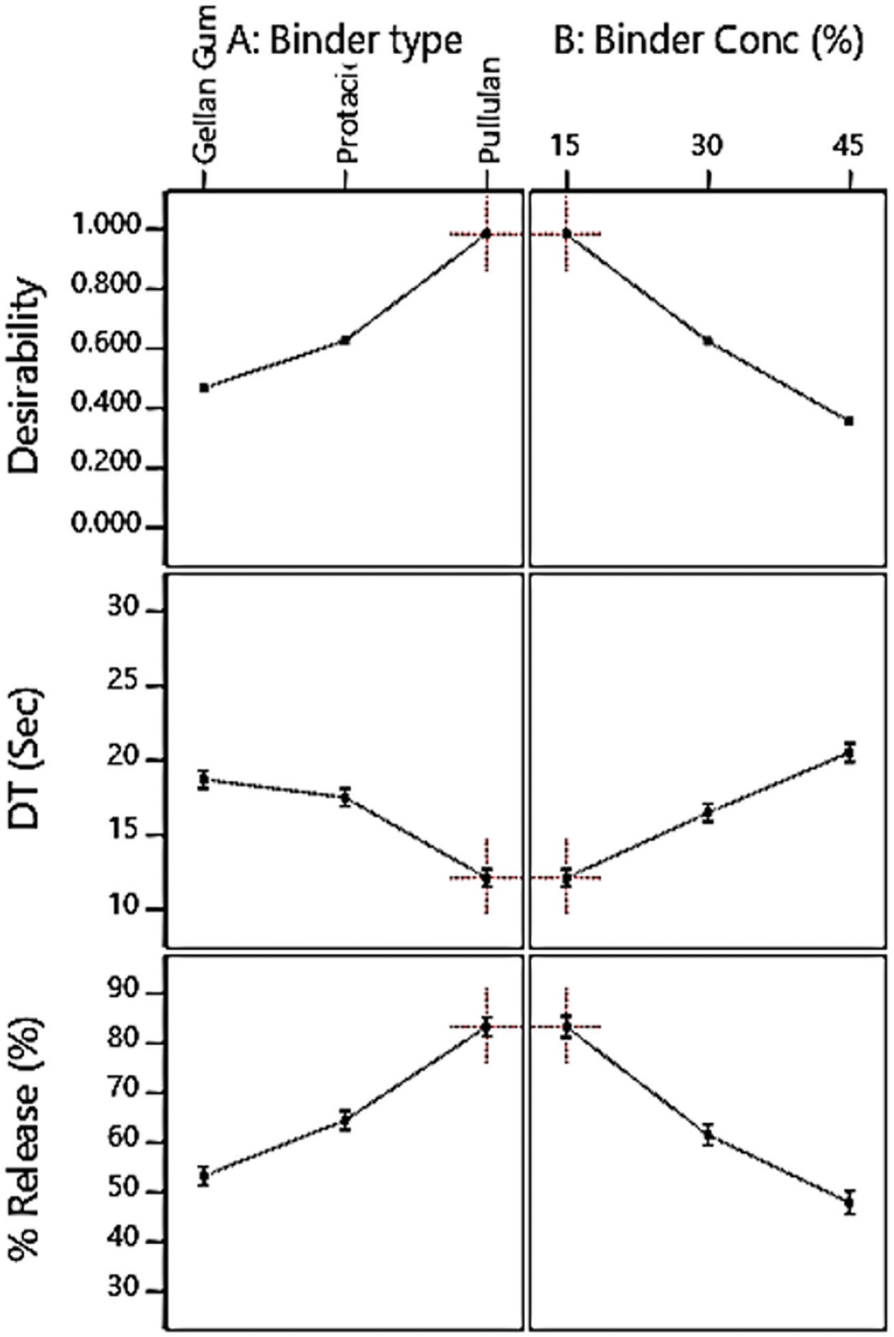
Influence of independent formulation variables (A) binder type and (B) binder concentration on DT and % SM release beside the overall desirability.

The disintegration of tablets is considered to be the rate-limiting step for the release of poorly water-soluble drugs (Tawfeek et al., 2020). Regarding DT results presented in Table 3, it was observed that all the prepared SM FMTs revealed rapid DT that ranged from 12.5 ± 0.64 to 28 ± 0.33 Sec and subsequently fell within the acceptable pharmacopeial limit for FMTs DT (FMTs should disintegrate within 30 Sec) (United States Pharmacopeia 24/NF19, 2000; ElMeshad et al., 2020; Tawfeek et al., 2020). The fastest DT was perceived for F7 FMT (12.5 ± 0.64 Sec) comprised a low level of binder concentration (15%) and pullulan as binder type as presented in (Figure 1). Additionally, the regression analysis showed that both the binder type and concentration had an obvious impact (*P* < .0001) on FMTs DT as shown in Table 4. Based on the sign of coefficient estimate for the effect of binder type and concentration (−4.00 and +4.37 respectively), the DT of FMTs was found to be negatively correlated with the former and positively with the latter. Figure 1 displayed that the DT of prepared FMTs was directly proportional to the binder concentration with an obvious increase in the DT at the level of a binder. Based on statistical data analysis for the effect of X_2_ on *in-vitro* DT (Table 4), it was observed that the binder concentration represents the most influential variable on FMTs DT, as evident by their sum of square values (117.10 for X_1_ and 164.27 for X_2_) and f-value (97.19 for X_1_ & 136.34 for X_2_) were increasing the level for X_2_ from low (15%) to high (45%) (F1-F3) resulted in slower DT where the former level recorded DT of 19 ± 0.25 Sec and the latter recorded 28 ± 0.33 Sec. Furthermore, the regression analysis showed that the binder concentration had a significant positive effect on DT of FMTs (*P* < .0001) corresponding to a positive sign of coefficient estimate (+4.37) as shown in (Table 4) indicating a positive correlation between X_2_ and DT. This could be attributed to the concentration raise of the binder in the FMT formulae led to an increase in the bond strength with the drug resulting in a reduction of pores surface, resulting in less tablet porosity and hence longer DT (Douroumis et al., 2011; ElMeshad et al., 2020). Another explanation is that when the number of binder fibers forming crosslinks increases, this might lead to the formation of a spacious and hard network with small pores after the lyophilization process (Ciper, and Bodmeier, 2005).

**Table 4.** Analysis of variance of SM-FMTs responses.

Variables	Coefficient estimates	Sum squares	*Df	Mean square	*F* (value)	*P* (value)
*DT						
Model	–	317.17	4	79.29	131.63	<.0001
*X_1_	−4.00	117.10	2	58.55	97.19	<.0001
*X_2_	4.37	164.27	2	82.13	136.34	<.0001
% SM release (Q_10_)						
Model	–	3054.63	4	381.83	184.71	<.0001
*X_1_	10.65	1070.60	2	535.30	258.95	<.0001
*X_2_	−12.63	1459.90	2	729.95	353.12	<.0001

*X_1_: binder type; X_2_: binder concentration; DT: Disintegration time; df: degree of freedom.

With respect to binder type, FMTs containing pullulan, as a binder, recorded the fastest DT (F7 = 13 ± 0.64 Sec) which could be attributed to its hydrophilic nature that allows quick erosion and the formation of pores filled with the solvent diffusing, causing the tablet to disintegrate faster (Cheng et al., 2011), while gellan gum recorded the highest DT (F3 = 28 ± 0.33 Sec) which could probably be explained by the high binding capacity of gellan gum and hence a more compact tablet (Emeje et al., 2010).

The regression equation is useful for identifying the relative impact of the factors by comparing the factor coefficients, it clarifies the influence of both X_1_ and X_2_ on DT is as follows:

$$Y_{1}=20.43-4.00\;X_{1}+4.37\;X_{2}$$

While the equations in terms of actual factors can be used to make predictions about the response for given levels of each factor. Actual Equations for DT are as follows:

$$Y_{1}\;\left(\text{DT}\right)=20.43+18.77\;A_{1}B_{1}+23.13A_{1}B_{2}+27.18A_{1}B_{3}$$

$$Y_{1}\;\left(\text{DT}\right)=20.43+17.56A_{2}B_{1}+21.92A_{2}B_{2}+25.97A_{2}B_{3}$$

$$Y_{1}\;\left(\text{DT}\right)=20.43+12.16\;A_{3}B_{1}+16.52A_{3}B_{2}+20.57A_{3}B_{3}$$

where, A_1_: Gellan Gum; A_2_: Protacid; A_3_: Pullulan and B_1_: 15%; B_2_: 30%; B_3_: 45%.

#### 3.1.3. *In-vitro* SM release from FMTs

**Figure 2.**
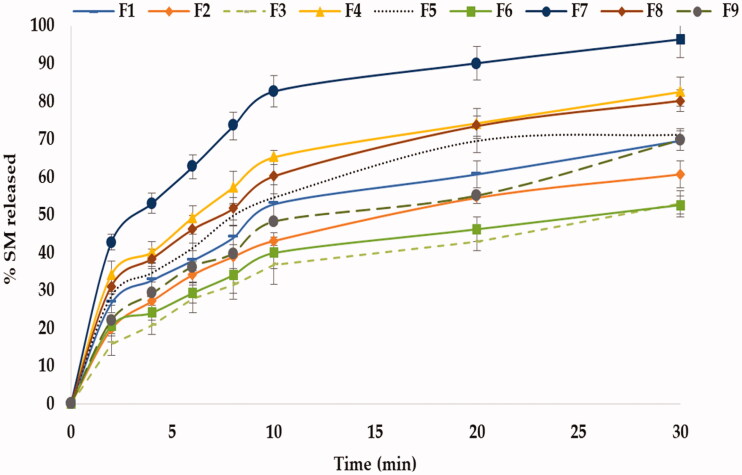
Schematic representation for *in-vitro* release of SM – FMTs.

Figure 2 illustrates the *in-vitro* release profile of SM from FMTs, demonstrating that all produced formulae exhibited rapid release profiles within 30 minutes, owing to the increased surface area available for dissolution following the tablets’ rapid disintegration into fine particles (Tawfeek et al., 2020). The SM-FMT formula containing 15% pullulan (F7) exhibited the highest release (Q_10_ = 83.40 ± 2.88%) as in Figure 2 which is correlated with the results of DT where F7 scored the lowest one (12.50 ± 0.64 Sec) and this falls with the concept that tablet disintegration is the rate-limiting step for drug release (Tawfeek et al., 2020). Statistical analysis showed that both binder type and concentration had a significant impact (*P* < .0001) on *in-vitro* SM release as shown in Table 4, where the coefficient estimate values demonstrated that the changes in the level of variable X_2_ had an obvious impact on SM release in an opposite direction based on evidence of both negative sign coefficient of estimate (−12.63) and the sum of squares (1459.90) respectively compared with variable X_1_ with a coefficient of estimate (+10.65) and a sum of squares (1070.60) (Table 4).

Figure 2 displayed the impact of X_1_ & X_2_ on % SM release where the release reduced upon increasing binder concentration from low to high level causing a sharp decline in % SM released from 65.28 ± 1.85% (15%) to 54.65 ± 5.31% (30%) and up to 39.98 ± 4.21% (45%). This could be attributed to the fact that decreasing the concentration of binder in FMTs led to an elevation in the porosity in the tablet matrix during the lyophilization process and thus enhance drug release (Ibrahim and El-Setouhy, 2010). Besides, a high level of binder caused tight binding between molecules which ultimately slows down the water uptake by the tablets and hence delays disintegration and drug release (Ibrahim and El-Setouhy, 2010). Another explanation is that, as the binder concentration was increased, the drug release decreased owing to the formation of a water-swollen gel-like structure that hinders the penetration and the dissolution medium into the matrix (Ahmed, 2018). The SM-FMTs formulated *via* pullulan at the same concentration (15%) recorded the highest SM release at Q_10_ (83.40 ± 2.88%) upon comparison with gellan gum (52.85 ± 5.24%) and protected (65.25 ± 1.85%) where a significant difference was observed (*P* < .0001) as shown in Table 4. These results have coincided with DT where pullulan had higher hydrophilicity which promotes water contact resulting in the disintegration of a tablet into small fragments hence increasing surface area causing higher SM release (Hosny et al., 2013; Tawfeek et al., 2020). The regression equation that clarified the impact of X_1_ and X_2_ on %SM release is as follows:

$$Y_{2}=53.70+10.65\;X_{1}-12.63\;X_{2}$$

While the actual equations for (%release):

$$Y_{2}=54.10+56.83\;A_{1}B_{1}+42.38\;A_{1}B_{2}+32.25A_{1}B_{3}$$

$$Y_{2}=54.10+66.06A_{2}B_{1}+51.61A_{2}B_{2}+41.48A_{2}B_{3}$$

$$Y_{2}=54.10+78.42\;A_{3}B_{1}+63.97A_{3}B_{2}+53.84A_{3}B_{3}$$

where, A_1_: Gellan Gum; A_2_: Protacid; A_3_: Pullulan and B_1_: 15%; B2: 30%; B3: 45%.

### 3.2. Determination of optimized formulation using desirability function

The criteria set for selection included attaining the minimum DT and maximum % SM released as depicted in Table 5, this was processed *via* the Design-Expert software to find the optimized formula with the desired attributes (AlAli et al., 2021). In the present study, the optimum values of independent variables were obtained using numerical optimization based on desirable conditions for all responses. It was found that the formulation prepared using a combination of a pullulan as binder type at a concentration of 15% achieved the required criteria with a higher desirability value of 0.987, as presented in Figure 1. Therefore, this formula was selected for further investigation.

**Table 5.** The Constraints implemented for the experiment variables in addition to the overall desirability.

Name	Goal	Lower limit	Upper limit	Lower weight	Upper weight	Importance
A: Binder type	In range	Gellan gum	Pullulan	1	1	–
B: Binder conc	In range	15	45	1	1	–
DT	Minimize	12	28	1	1	+++++
% SM release (Q_10_)	Maximize	34.17	84.11	1	1	+++++

#### 3.2.1. Fourier transform infrared spectroscopy (FT-IR)

**Figure 3.**
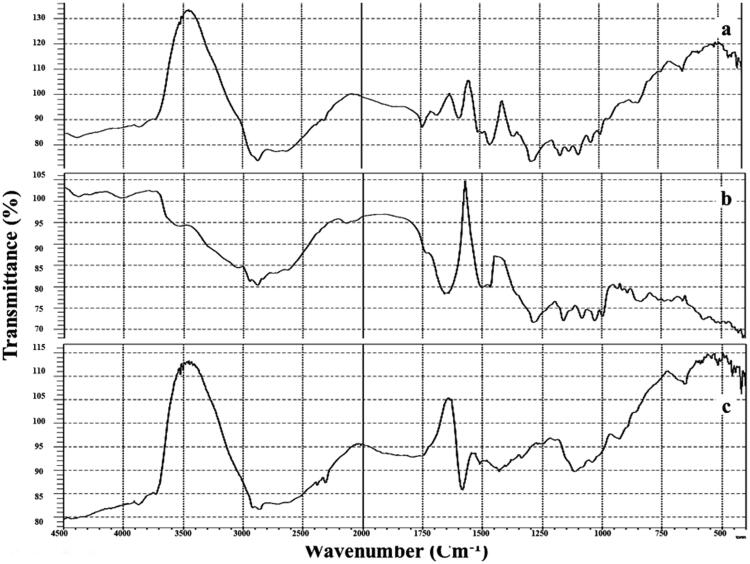
FT-IR of: (a) Free SM, (b) Plain F7, and (c) F7.

The pure SM, plain F7 and F7 FT-IR spectra are displayed in Figure 3, SM showed its main peaks at 1600 cm^−1^ attributed to carbonyl vibrations; 1450 cm^−1^ reckoned to the symmetric aromatic ring stretching; 1045 cm^−1^ referred to the benzopyran ring with the simultaneous presence of out plane -CH deformations at 845 cm^−1^. FT-IR spectra of the F7 showed the characteristic peaks without shifting, thus indicating the absence of interaction between SM and excipients.

#### 3.2.2. X-Ray powder diffraction (XRD)

**Figure 4.**
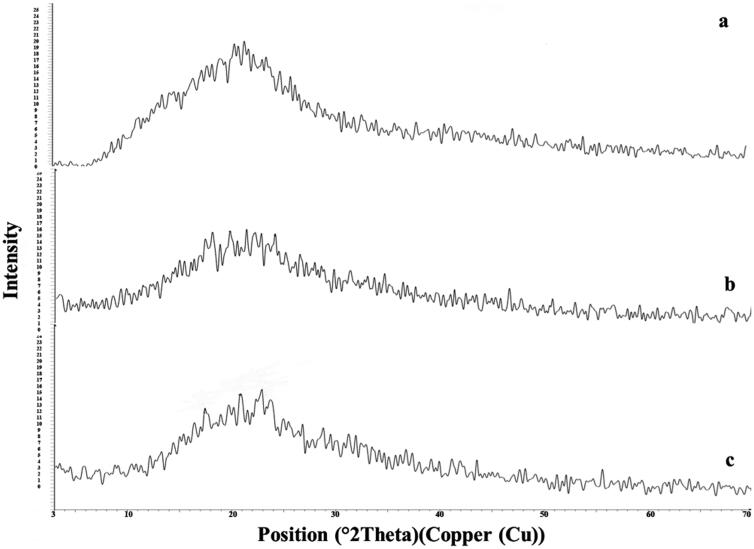
X-ray scans of: (a) Free SM, (b) Plain F7, and (c) F7.

The x-ray of SM, plain F7, and F7 are represented in Figure 4(a–c). The diffractogram of pure SM showed intense peaks indicative of its crystallinity. In the case of plain F7, a low crystallinity was observed. However, in the case of the F7 diffractogram, a reduced number of signals, of markedly low intensity were noticed, indicating the inclusion of SM inside the ODT form (more water-soluble) (Sheta et al., 2020; Soliman et al., 2020).

#### 3.2.3. Scanning electron microscopy (SEM) visualization

**Figure 5.**
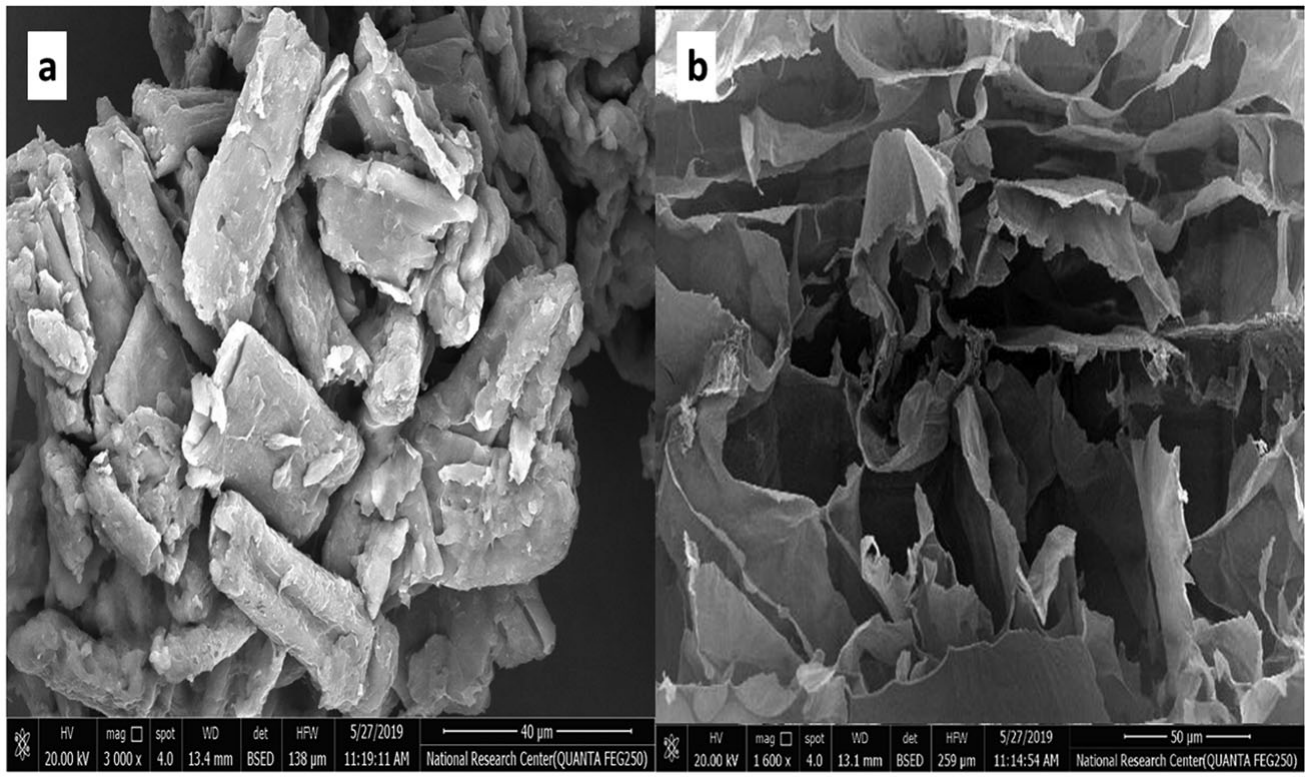
Scanning electron microscope of (a) free SM and (b) optimized F7 FMT containing SM.

Scanning electron micrographs of SM and the cross-section view of F7 are displayed in Figure 5. SM micrograph revealed a regular crystalline surface of particles. The micrograph of F7 shows large, deep, and diffusible pores that form channels through which the dissolution medium penetrates, facilitating tablet disintegration, which might elucidate the faster *in-vitro* disintegration and drug dissolution (Ibrahim and El-Setouhy, 2010; ElMeshad et al., 2020).

### 3.3. Investigation of lung-protective efficacy of SM and F7 in HgCl_2_ inducing lung toxicity in rats

Table 6, revealed an apparent elevation in plasma TNF-α and TGF- β (*P <* .05) in the second group which represents the HgCl_2_ (1 mg/kg) treated rats when compared to the normal control group. The uptake of SM and F7 clarified a significant decrease in TNF-α, as well as TGF-β values relative to the HgCl2, treated group of rats after 15 days (*P <* .05) with better results concerning TNF-α in F7.

**Table 6.** Effect of SM FMTs and free SM on plasma TNF-α and TGF-β in rats.

No.	Groups	TNF-α (pg/mL)	TGF-β (pg/mL)
(I)	Normal (5 mL distilled water)	27.60 ± 4.76^a^	89.28 ± 7.48^a^
(II)	Positive control HgCl_2_ (1 mg per kg.bw per day) in distilled water	188.09 ± 10.02^b^	104.34 ± 5.85^b^
(III)	HgCl_2_ (1 mg per kg) + F7 (100 mg per kg)	31.89 ± 2.83^c^	81.96 ± 10.49^c^
(IV)	HgCl_2_ (1 mg per kg) + free SM (100 mg per kg)	36.21 ± 2.17^d^	60.66 ± 13.75^d^

Data shown represent mean ± standard deviation, (*n* = 10). Data followed by different letters within the same parameter are significantly different at *P* ≤ 0.05.

Mercury is one of the most common heavy metals used in agriculture, pharmacology, and industry (Agha et al., 2014). Heavy metals enter the human body and produce significant poisoning (Alissa and Ferns, 2011). HgCl_2_ in biological tissues induces a number of negative changes that have an effect on the organism’s health (Othman et al., 2014). Heavy metals are persistent and common contaminants that cause oxidative stress, which disrupt the structure and function of many organs. Many reactive oxygen species such as superoxide and hydrogen peroxides are enhanced by mercury compounds causing lipid peroxidation and oxidative tissue harm (García-Niño and Pedraza-Chaverri, 2014). The present study clarified that HgCl_2_ can result in an obvious elevation in the plasma level of TNF-α and TGF-β, indicating inflammation. Previous studies have demonstrated those heavy metals caused elevated TNF-α and TGF-β levels in the lung tissue (Cuneyt et al., 2019).

Natural products treatment may be considered a viable strategy to reduce inflammation. SM may help to prevent the negative effects of inflammation reactions. The molecular and cellular defensive protective mechanisms of SM include either free radicals scavenging or cellular structure protection such as membrane and lipid domain and regulating particular enzymes. It may also stop the generation of cytokines (Fernandez, and Eickelberg, 2012). Several investigations have clarified that SM can resist tissue and organ damage induced by heavy metals (Surai, 2015).

It may exert its effects *via* altering cell signaling pathways, which are mostly activated due to oxidative and inflammatory stimuli. Additionally, Nrf2 and NF-κB are the principal downstream effectors (Gessner et al., 2013). The lung-protective action of F7 is stronger than free SM. FMT synchronous delivery, therefore, is important and exerts lung protection activity in case of HgCl_2_ overdose.

Table 7 shows a significantly increased (*P <* .05) IL-6 as well as lung caspase 3 and MCP-1 in the HgCl_2_ treated rats compared with the normal control group, conducting acute lung inflammation. Free SM and F7 treatment significantly decreased plasma IL-6 as well as lung caspase 3 and MCP-1, as compared with the HgCl_2_-treated group (*P <* .05). Our results indicated that F4 treatment showed better improvement in IL6, caspase 3, and Mcp-1 than free SM.

**Table 7.** The influence of SM FMTs and free SM on plasma IL-6 as well as lung caspase 3 and MCP-1 in rats.

No.	Groups	Plasma IL-6 (pg/mL)	Lung caspase 3 (pg/mL)	Lung MCP-1 (pg/mL)
(I)	Normal (5 mL distilled water)	3.22 ± 0.59^a^	5.00 ± 0.89^a^	128.79 ± 13.45^a^
(II)	Positive control HgCl_2_ (1 mg per kg per day) in distilled water	14.61 ± 2.18^b^	18.55 ± 2.87^b^	470.92 ± 17.90^b^
(III)	HgCl_2_ (1 mg per kg) + F7 (100 mg per kg)	4.99 ± 0.09^c^	7.92 ± 1.53^c^	153.20 ± 10.55^c^
(IV)	HgCl_2_ (1 mg per kg) + free SM (100 mg per kg)	9.36 ± 1.33^d^	10.55 ± 2.95^d^	203.51 ± 16.07^d^

Data shown represent mean ± standard deviation, (*n* = 10). Data followed by different letters within the same parameter are significantly different at *P* ≤ .05.

One possible mechanism could be that HgCl_2_ causes IL-6 and MCP-1 to translocate into the nucleus, hence controlling interactions of IL-6 with pregnane X retinoid X receptor complex (Smetana and Brábek, 2020). In keeping with these findings, we found that the levels of pro-inflammatory cytokines (IL-6, MCP-1, and Caspase-3) were dramatically enhanced after HgCl_2_ exposure. Lung destruction is associated with cytokine storm-related to elevate IL-6 levels (Smetana and Brábek, 2020). Inhibition of the interaction between MCP 1 and CCR 2 (Chemokines receptor 2) can alleviate acute lung injury in rats (Cao et al., 2018). Caspase 3 was elevated in altered lung morphologies (Fodor et al., 2016).

**Figure 6.**
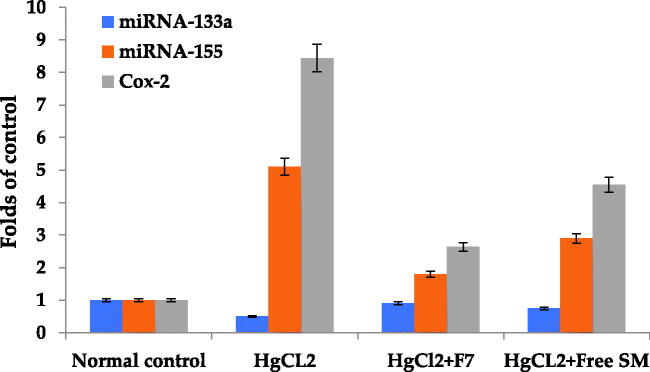
Effect of free SM and F7 on lung miRNA 133a, 155 expression and Cox-2 protein expression in rats. Data (*n* = 10 per group) are presented as folds of increase or decrease compared to the control supposing the control value is one.

Expression of miR-133-a was significantly lower (*P <* .05) in HgCl_2_ (1.0 mg/kg) treated rats than in the normal control group. Additionally, free SM and F7 treatment significantly increased (*P <* .05) miR-133-a expression in the lungs of rats compared to the HgCl_2_-treated group with better results in F7 (Figure 6). Expression of miR-155 was considerably greater (*P <* .05) in HgCl_2_ (1.0 mg/kg) treated rats compared to the normal control group. Moreover, free SM and F7 treatment significantly suppressed (*P <* .05) miR-155 expression in the lung of treated rats as compared with the HgCl_2_-treated group (Figure 6) with better improvement in F7.

**Figure 7.**
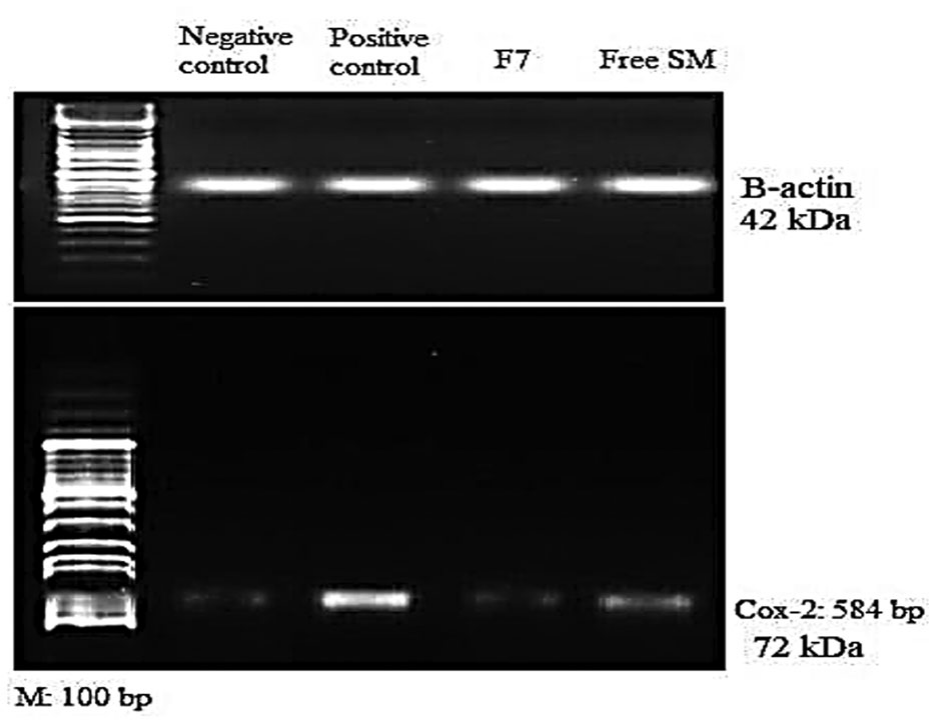
Agarose gel electrophoresis showing western blot quantification of lung COX-2 protein expression and beta-actin treated rats, I: (Normal control); II: (HgCl_2_ Group); III: (HgCl_2_ + F7 Group) and IV: (HgCl_2_ + Free SM Group).

Figure 6, shows that HgCl_2_ (1 mg/kg) enhanced the COX-2 protein expression in the HgCl_2_-treated group of rats compared to the control group (*P <* .05). Also, administration of free SM and F7 treatment resulted in a significant reduction in COX-2 expression compared to the HgCl_2_ treated group (*P <* .05). Images of agarose gel electrophoresis of COX-2 protein expression and β-actin supported the presented results, Figure 7.

Previous studies revealed that miRNA −155 was decreased in bronchial biopsies from chronic obstructive pulmonary disease (COPD) patients treated with corticosteroids for 6 or 30 months (Boateng and Krauss-Etschmann, 2020). Francis *et al.* (2014) [65] found five miRNA (miR-34a, miR-34b, miR-133a, miR-133b, and miR-149) were down-regulated in advanced emphysema (Francis et al., 2014). Recent evidence clarified that SM might protect against tissue damage caused by carbon tetrachloride, and HCl (Rasha et al., 2019). In line with earlier outcomes, our data demonstrated that SM reduced HgCl_2_-induced apoptosis and oxidative. In terms of the mechanisms behind SM protective benefits against HgCl_2_-triggered liver toxicity, we found that SM markedly inhibited COX-2 protein expression, and decreased cytokines levels compared with the HgCl_2_ groups.

HgCl_2_ administration in the present study up-regulated COX-2 protein, which is consistent with a previous study (Gust et al., 1999). The inducible COX-2 is an inflammatory marker, which when blocked by parenteral SM can attenuate the inflammatory response and oxygenation impairment after HCl aspiration (Terao et al., 2005). COX-2 is found in resident inflammatory cells in the lung, as well as the pulmonary endothelium and epithelium, and its activation causes an increase in the expression of prostanoids, which are important mediators of lung inflammation (Robertson et al., 2012). SM in this study significantly decreased COX-2 protein confirming its anti-inflammatory activity that was previously reported (El-Lakkany et al., 2012). SM has been shown to minimize COX-2 expression in various tissues (Sharifi et al., 2013). Additionally, F7 showed better improvement concerning COX-2 expression as compared with free SM.

**Figure 8.**
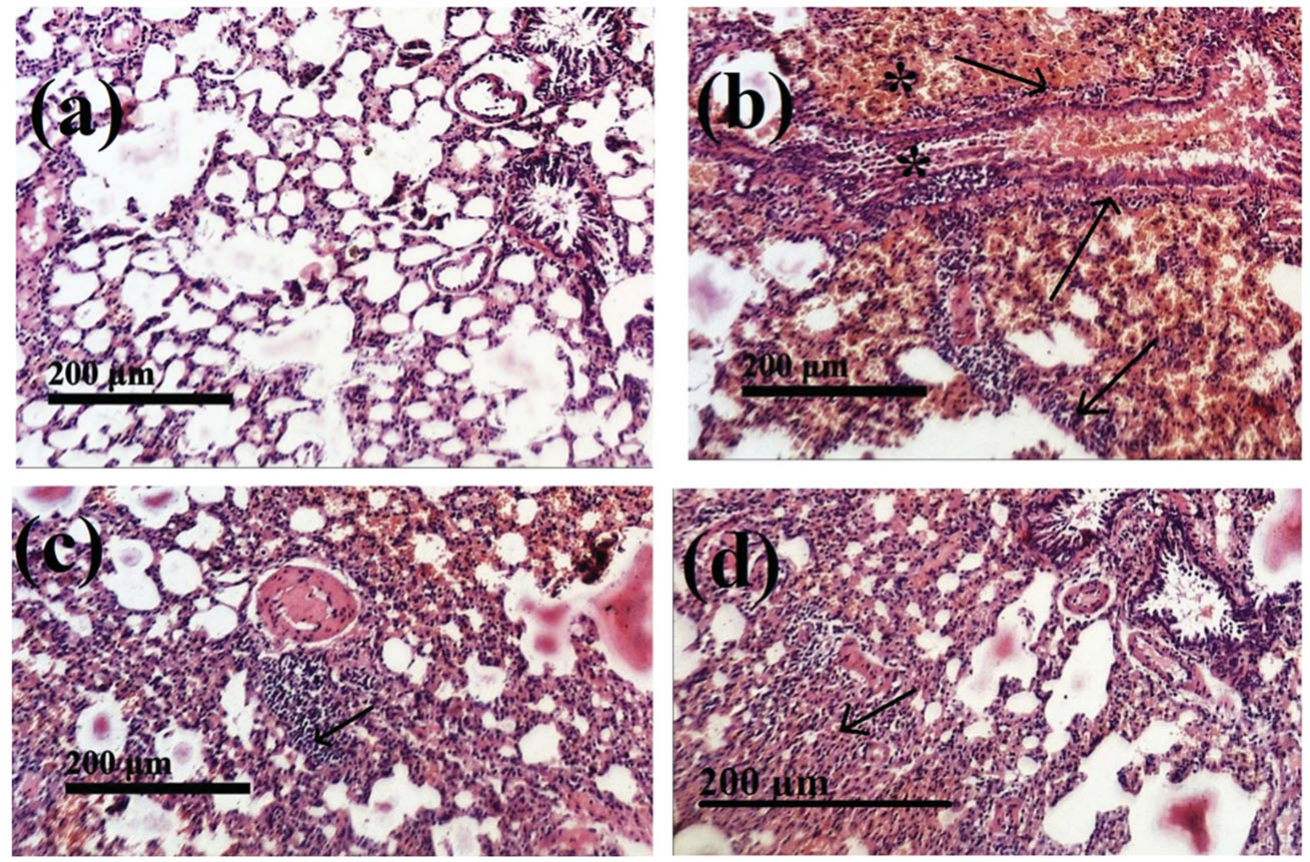
The stained (H&E; 200 X) histological sections examination of rats lungs of different groups compared to control group, (a): Negative control group (I); (b): Group II (Positive control) received HgCl_2_ (1 mg/kg) for 15 days; (c): Group III was treated with HgCl_2_ (1 mg/kg) + F7 (100 mg/kg) for 15 days (d); Group IV was treated with HgCl_2_ (1 mg/kg) + Free SM (100 mg/kg) for 15 days.

Histopathological examination of lung slices from the normal group (I) showed normal morphological features of lung parenchyma with apparent intact respiratory airways epithelium as well as alveolar walls (arrows) with intact vasculatures Figure 8(a). Conversely, in the lung of the HgCl_2_-treated control group (II), histological examination revealed marked diffuse hemorrhagic pneumonia with extravasation of blood into lumen alveoli and intrabronchioles accompanied by severe thickening of the walls of interalveolar and peribronchiolar tissue with infiltrates of inflammatory cells (arrow) Figure 8(b).

Histopathological investigation revealed good recovery from HgCl_2_-induced lung toxicity by F7 (group III) compared to the HgCl_2_-treated group and clarified almost the same records as Groups I, Figure 8(c). In group (IV) all samples of HgCl_2_ treated rats were improved by free SM (100 mg/kg) administration showing intact morphological features of pulmonary tissue with reduced records of infiltrates of inflammatory cells with almost the same records as group III, Figure 8(d).

## 4. Conclusion

SM-FMTs were efficaciously formulated *via* lyophilization technique employing gellan gum, protacid, and pullulan as binders with different levels (15, 30% 45%) where all prepared formulae possess acceptable rapid disintegration and instant SM release. Based on criteria settled for a factorial design, F7 FMT was formulated using a low level of pullulan (15%) and scored the fastest DT of (12.50 ± 0.64 Sec) and highest Q_10_ of (83.40 ± 2.88%) and was denoted as the optimized formula with desirability value of 0.987, which was evaluated in term of lung toxicity protection. F7 upregulated miRNA133a to nearly two-fold and downregulated miRNA155 and COX-2 protein expression by about 47% and 66% respectively, it also caused a decrease in cytokines TNF-α, TGF-β, IL-6, caspase 3 and MCP-1 levels by 81%, 42%, 36%, 43%, and 57% respectively compared to the positive control, most of the studied parameters were improved in F7 compared to free SM. Consequently, SM-FMTs formulation could be considered as an alternative to the marketed product as a result of combining the advantages of rapid onset of action and eradicating the problem of swallowing complications, particularly in elderly individuals. Further studies could be performed to investigate the lung protection for other injuries e.g., post covid, besides further pharmacokinetic clinical studies to support the obtained results.

## Data Availability

All relevant data are within the manuscript and any other additional materials are available upon request.
